# The role of T Cell Immunoglobulin Mucin Domain-3 in children with immune thrombocytopenia

**DOI:** 10.1186/s12887-026-06657-1

**Published:** 2026-03-24

**Authors:** Aya Ali Hamam, Ibrahim Mohamed Badraia, Hossam Abd El Mohsein Hodeib, Hend Mohamed Shamhout

**Affiliations:** 1 Pediatric Department, Faculty of Medicine, Al Azhar University, Cairo, Egypt; 2 Pediatric Department, Faculty of Medicine, Tanta University, Tanta, Egypt; 3 Clinical Pathology Department, Faculty of Medicine, Tanta University, Tanta, Egypt

**Keywords:** T-Cell, TIM-3, Immune Thrombocytopenia, Immunoglobulin

## Abstract

**Background:**

Immune thrombocytopenia (ITP) is an immune-mediated disorder marked by impaired self-tolerance, leading to accelerated platelet destruction, reduced platelet production, and a range of bleeding manifestations. This study aimed to evaluate the role of TIM-3 in children with newly diagnosed ITP.

**Methods:**

This cross-sectional study included 80 children, comprising two equal groups. Group I consisted of children with newly diagnosed primary ITP, from whom samples were collected at diagnosis before treatment and again at remission (platelet count ≥ 100 × 10⁹/L with resolution of bleeding symptoms after treatment). Group II included age, and sex matched healthy children serving as controls.

**Results:**

TIM-3 levels were significantly and positively correlated with platelet counts at both diagnosis and remission (*P* < 0.05), while showing a significant negative correlation with bleeding scores (*P* ≤ 0.001). Hemoglobin levels increased markedly after remission compared with diagnosis. White blood cell counts at diagnosis differed highly significantly between patients and controls (*P* ≤ 0.001), and TIM-3 levels at diagnosis were significantly lower in the ITP group than in healthy controls.

**Conclusions:**

This study assessed TIM-3 role in newly diagnosed children with ITP. According to the results, TIM-3 could affect the immune response and platelet destruction in individuals with ITP, suggesting a key function for this protein in the disease’s pathophysiology.

**Trial registration:**

Not applicable.

## Background

The autoimmune illness known as immune thrombocytopenia (ITP) is characterized by a low platelet count in peripheral blood (< 100 × 10^9^/L) that any other medical conditions cannot explain. less Multiple pathways contribute to immunological dysregulation, which in turn causes ITP. Important roles in the pathogenesis of ITP are played by these systems, which include complement fixation, malfunction of T lymphocytes, and the production of immunoglobulin G (IgG) type antiplatelet antibodies against many platelet surface antigens [1]. A low platelet count, caused by an increase in platelet breakdown and a decrease in platelet formation, is a hallmark of ITP. Its pathophysiology is complex and involves significant abnormalities in T-lymphocytes. Key immune mechanisms include a Th1-dominant response, impaired function or reduced numbers of regulatory T cells, as well as direct destruction of platelets by cytotoxic (CD8+) T cells. All these disruptions point to how vital T-cell dysregulation is for ITP onset and maintenance [2]. In ITP, T-lymphocyte dysregulation plays a key role. This includes a shift in the Th1/Th2 balance toward Th1 dominance, an increase in Th17 cells, and a decrease in the number of CD4^+^ CD25^+^ regulatory T cells, which generally help suppress excessive T-cell responses. Another factor that destroys platelets is cytotoxic CD8 + T lymphocytes, as elevated levels of platelet-specific CD8^+^ cells are observed in active ITP. Additionally, the CD4^+^:CD8^+^ ratio is decreased during active disease and tends to normalize with remission. [3]. There is a lack of long-term efficacy in many therapies for autoimmune diseases. There has been a change in emphasis towards enhancing health-related quality of life and alleviating symptoms in many autoimmune illnesses. The same holds true for ITP, where improving patients’ quality of life is one of the primary aims of therapy [4].

Bruises and petechial rashes are the usual early symptoms of the condition, which affects almost every patient. Approximately one-third of individuals may experience nosebleeds, while hematuria is relatively uncommon. In most cases, symptoms appear about two weeks after a viral upper respiratory infection. Despite visible signs, affected children usually appear well and exhibit no organ enlargement or lymph node swelling [5]. In children, ITP usually strikes a young, otherwise healthy youngster between the ages of two and seven. Both sexes are equally impacted [6]. Among the several types of primary ITP, there are three distinct time intervals between diagnoses: newly diagnosed (within three months), persistent (between three and twelve months), and chronic (more than twelve months) [4].

The first line of defense against newly diagnosed pediatric ITP might be observation alone, intravenous immunoglobulin (IVIG), steroids, anti-D immunoglobulin, or a mix of these therapies. Some research suggests that splenectomy may help children with chronic ITP. A chimeric monoclonal antibody (anti-CD20) called rituximab may postpone splenectomy and even cause complete remission in certain patients. Romiplostim and Eltrombopag, two thrombopoietin (TPO) agonists, have recently shown promising results in treating severe chronic ITP in both adults and children [7]. One protein that aids in controlling immunological responses is TIM-3. Initially known for suppressing Th1 cells through galectin-mediated cell death, its role has expanded to include promoting immune tolerance via T-regs, contributing to immunosuppression in Th17 and CD8^+^ T cells, and modulating innate immune cell functions [8].

This work aimed to evaluate TIM-3 role in newly diagnosed children with ITP.

## Methods

This cross-sectional study involved 80 children, ranging in age from 1 to 18 years old and of both sexes, with newly diagnosed primary ITP and a platelet count of less than or equal to 30,000/µL (or more than or equal to 30,000/µL with evidence of mucus hemorrhage). The international consensus guidelines for the diagnosis of ITP state that the patient must meet the following criteria: two blood tests showing platelet counts below 100 × 10^9^/L; standard shape of blood cells; obvious signs of bleeding in the skin, mucous membranes, or organs; absence of splenomegaly; and the exclusion of other types of secondary thrombocytopenia, such as aplastic anemia or inherited thrombocytopenia caused by other immune diseases, infections, or drugs [9].

The study was conducted with approval from the Ethical Committee at Tanta University Hospitals, Tanta, Egypt. This study was done in accordance with the Declaration of Helsinki. Informed written consent was obtained from the patients or their relatives. An informed written consent was obtained from all patients and their relatives.

Children with secondary thrombocytopenia resulting from leukemia, hepatitis C, drugs, lupus erythematosus, cirrhosis, antiphospholipid syndrome, von Willebrand factor deficiency, and congenital thrombocytopenia were not eligible to participate in the study.

Two equal groups of patients were categorized: Group I: children with newly diagnosed ITP (At the time of diagnosis, samples were collected from this particular group (before receiving any treatment) and at remission time (platelet count of ≥ 100 × 10^9^ with concurrent resolution of bleeding symptoms after receiving treatment) [10] and Group II: children of the same age and gender who are in good health.

Complete medical histories were taken from all patients and tested for various conditions and abnormalities, including a complete blood count (CBC), erythrocyte sedimentation rate (ESR), C-reactive protein, liver and kidney function tests, and a bone marrow aspiration. The physical examinations included vital signs like blood pressure, temperature, heart rate, and respiratory rate, as well as signs of illness like pallor, cyanosis, jaundice, and enlarged lymph nodes, complete physical examination: encompassing degree of bleeding symptoms, abdominal and lymph node examination, score of bleeding and specific investigation [11].

### Intended use

This kit was designed for the quantitative in vitro assessment of MUC3 in various biological fluids, including cell lysates, human tissue homogenates, cell culture supernates, and other similar samples. It was a sandwich enzyme immunoassay.

### Assay procedure

A diluted standard, a blank, and a sample were used to determine the wells. One well was set aside as a blank, while seven wells were readied for the standards. 100µL of each dilution of the standard (see Reagent Preparation), blank, and samples were added to the corresponding wells. The sealer for the plate was covered. Holding at 37 °C for a duration of two hours. Do not wash the wells; the liquid has been drained. Detection Reagent measuring 100µL. Each well has a functional solution added to it. Please place it in an incubator set at 37 °C for one hour after applying the plate sealer. Using a squirt bottle, multi-channel pipette, manifold dispenser, or auto-washer, 300µL of 1× Washing Solution was added to each well to rinse the solution, and it was allowed to settle for one to two minutes. By tapping the plate into absorbent paper, all of the leftover liquid was evacuated from the wells. After that, wash it well three times. Nothing remained after the last wash. Use an aspirator or a decanter to rinse the buffer. A piece of absorbent paper was used to wipe the plate after it was inverted. Each well received 100 µL of the working solution of Detection Reagent B. After sealing the plate, it was placed in the incubator for one hour at 37 °C. Following the steps outlined in step 4, the aspiration/wash procedure was performed five times. Each well was supplemented with 90µL of Substrate Solution. A brand-new plate sealer was dressed. Incubating at 37 °C for 15 to 25 min (no more than 30 min) and following that, shielding against light. When the Substrate Solution was added, the liquid changed color to blue. A total of 50 µL of Stop Solution was added to each well. After adding the Stop solution, the liquid became yellow. To combine the ingredients, smack the plate on the work surface. If the shift in color doesn’t seem uniform, gently tap the plate to ensure everything is well mixed. There were no visible bubbles in the liquid, and fingerprints on the plate’s underside verify this, as well as the removal of any water droplets. The microplate reader was started, and readings were taken immediately at 450 nm.

### Statistical analysis

For statistical analysis, data were input into a computer using the SPSS (Statistical Package for Social Science) application version 21.0, developed by SPSS Inc. of Chicago, Illinois, USA. Numbers or categories were used to input the data. We displayed quantitative data as mean, standard deviation, and range. The frequency and percentage of qualitative data were presented with a 95% confidence interval (95% CI). To assess the degree of correlation between two groups of qualitative variables, we employed chi-square and Fisher’s exact tests. The means and standard deviations of two sets of quantitative data were compared using the Student t-test and the Mann-Whitney test, respectively, to determine whether the data were normally distributed. The correlation coefficient (r) was used in correlation analysis to compare the correlation between two continuous variables. A P (probability) value below 0.05 was considered statistically significant.

## Results

Regarding age, sex, and hemoglobin level at diagnosis, none of the evaluated groups showed significant differences. The platelet count and TIM-3 level in the case group were significantly lower than those in the control group at the time of diagnosis. After remission, the platelet count and hemoglobin level of the case group were significantly higher than they had been at the time of diagnosis. Furthermore, at the time of diagnosis, there was a significantly different white blood cell (WBC) count in the case and control groups (Table 1).

**Table 1:** Demographic and laboratory data between the studied groups

			Group I (n=40)		Group II (n=40)	*P*
Age			6.47±2.18		7.3±3.8	0.23
Sex	Male		26(65%)		18(45%)	0.07
	Female		14(35%)		22(55%)	
Platelet count						
At diagnosis		17.5(11.75-30)		202.5(176.25-278)		≤0.001*
At remission		100(80-147.5)		150(90-202.5)		0.342
P_1_		≤0.001*		0.808		
Hemoglobin level						
At diagnosis		11.25±1.21		11.7±1		0.07
P_1_		≤0.001*		0.06		
WBCs count						
At diagnosis		11.9±6.22		7.34±1.48		≤0.001*
P_1_		≤0.001*		0.95		
TIM-3 level		240(84.7- 2419.9)		765(113.2- 1890.8)		≤0.001*

Data are presented as mean ± SD or frequency (%) or median (IQR). *WBCs*: White Blood Cells. *: Statistically significant at *P* ≤ 0.05. *P*: Compare between group I and group II, P1: Compare between groups at diagnosis and at remission

Patients in remission did not vary significantly from healthy controls with respect to TIM-3 level or platelet count (Table 2).

**Table 2:** Comparison between TIM-3 level and platelet count in patients at remission and healthy controls

	Group I at remission(n=40)	Group II (n=40)	*P*
TIM-3 level at remission (ng/l)	2536 (2149 - 3132.75)	2767 (2381 - 3295.75)	0.148
Platelet count at remission	100 (80-147.5)	150 (90-202.5)	0.342

Data is presented as median (IQR)

The most common symptom of bleeding was purpura, accounting for 37.5% of cases, followed by ecchymosis and bleeding from the gums 30% and 20% of cases. Other symptoms, such as epistaxis (10%), were less frequent, while hematochezia was observed in only 2.5% of cases. The score of bleeding reveals that most cases (67.5%) fall within the moderate severity category (score 3), while 12.5% have mild bleeding (score 2) and 20% present with severe bleeding (score 4). Skin bleeding is the most common (67.5%), followed by bleeding from the mouth (20%), nose (10%), and rectum bleeding being the least common of cases (2.5%) (Table 3).

**Table 3:** Clinical assessment of case group

		Group I (n=40)
Clinical presentation	Purpura	15(37.5%)
	Ecchymosis	12(30%)
	Bleeding per gum	8(20%)
	Epistaxis	4(10%)
	Hematochazia	1(2.5%)
Score of bleeding		
Moderate	27(67.5%)	
	High risk	12(44.44%)
	Low risk	15(55.56%)
Severe		8(20%)
Mild		5(12.5%)
Site of bleeding	Skin	27(67.5%)
	Mouth	8(20%)
	Nose	4(10%)
	Rectum	1(2.5%)

Data is presented as frequency (%)

At both diagnosis and remission, a significant positive association was found between TIM-3 level and platelet count (*P* < 0.05). The amount of TIM-3 was negatively correlated with the bleeding score (*P* ≤ 0.001) (Table 4).

**Table 4:** Correlation between TIM-3 level with different parameters at diagnosis, remission and score of bleeding

		TIM-3 level	
		*r*	*P*
**At diagnosis**	Platelet count	0.479	0.001*
	Hemoglobin level	0.432	0.005*
	WBCs count	0.372	0.018*
**At remission**	Platelet count	0.667	< 0.001*
	Hemoglobin level	-0.229	0.04*
	WBCs count	0.062	0.587
**Score of bleeding**		-0.776	˂0.001*

*r* correlation coefficient, *WBCS* White Blood Cells. *: Statistically significant at *P* ≤ 0.05

## Discussion

In ITP, abnormalities in immunological self-tolerance cause an increased rate of platelet destruction and a decrease in platelet synthesis, creating a thrombocytopenic condition characterized by various bleeding symptoms [12].

Purpura was the most common bleeding manifestation (37.5%), followed by ecchymosis (30%) and gum bleeding (20%), while epistaxis (10%) and hematochezia (2.5%) were less frequent. Most patients had moderate bleeding severity (67.5%), with skin bleeding being the predominant site, followed by oral, nasal, and rarely rectal bleeding.

In the same line as Kasamatsu et al. [13] found that symptoms of bleeding were significantly higher in the case group than usual, such as purpura, ecchymosis, and epistaxis.

At diagnosis, platelet counts were significantly lower in the ITP group than in controls, while during remission, patients showed a significant increase in platelet counts compared with levels at diagnosis.

In the same line as Kasamatsu et al. [13] found that there was a significantly lower level in the case group than the control group regarding platelet count.

The study showed no significant difference in hemoglobin levels between groups at diagnosis or remission; however, hemoglobin levels differed significantly between diagnosis and remission within the case group, with no significant change in controls. A statistically significant difference in white blood cell counts was observed between cases and controls at diagnosis.

In supporting Hussein and Al-Mousawi [14] found the mean WBC count in ITP patients (7.642 ± 3.030) was comparable to that of healthy controls (7.243 ± 1.643), with no significant overall difference. Although acute ITP cases showed a slightly higher WBC count (8.533 ± 3.698) than chronic cases (7.362 ± 2.785), this difference was minimal, reflecting mild inflammatory changes associated with the disease.

According to the distribution of serum TIM-3 level between the studied groups, there was a statistically significantly lower level in the case group than the control group (median 240 (84.7-2419.9) ng/l in the case group at diagnosis), (median 765 (113.2-1890.8) ng/l in the control group).

The newly diagnosed patients had a significantly lower proportion of serum TIM-3, according to Sun et al. [2] (mean ± SD 0.7767% ± 0.1126%; *N* = 26; *P* < 0.01). *P* < 0.01; newly diagnosed ITP patients had a significantly reduced serum TIM-3% compared with remission patients (mean ± SD, 1.578% ± 0.6121%).

This study demonstrated a positive correlation between TIM-3 levels and platelet counts at diagnosis and during remission in ITP patients. Lower platelet counts were associated with reduced TIM-3 levels, suggesting that TIM-3, an immune regulatory protein, may play a role in the immune response to platelet destruction in ITP.

Since TIM-3 acts as a negative regulator of effector T cells, its downregulation in CD4⁺ T cells may contribute to ITP development and progression. Restoring TIM-3 expression could correct immune dysregulation, suggesting a potential new target for immunotherapy in ITP.

They offered evidence that, according to Gorman and Colgan [17], TIM-3’s function may change under different conditions. Our existing data that TIM-3 may activate T cells under circumstances involving acute stimulation is expected to be further strengthened by more research on TIM-3. Research using mice has shown that naïve CD8 T cells do not express Tim-3, whereas activated CD8 T cells caused by microbial infection do. Furthermore, several investigations in mice or humans have shown that CD8 T cell activation leads to elevated Tim-3 expression during persistent infections.

We also demonstrated a comparison between TIM-3 level and platelet count in patients at remission and healthy controls and revealed that there was no statistically significant difference in TIM-3 level and platelet count in patients at remission compared to healthy controls.

In contrast with Elfiky et al. [15], who reported a significant negative correlation between TIM-3 level, platelet count, and hemoglobin level.

We found a correlation between TIM-3 level and the score of bleeding; specifically, there was a significant negative correlation between TIM-3 level and the bleeding score.

Patients with ITP exhibited lower levels of TIM-3 expression in CD56 + high NK cells, with the lowest levels observed in those with severe bleeding, as reported by Zahran et al. [16] It was proposed that TIM-3 expression is directly correlated with illness severity based on this data.

The study suggested that further research with larger samples should be conducted to determine the function of TIM-3 in newly diagnosed ITP children and its relationship to the course of the disease. It also suggested that flow cytometry and other methods should be used to learn more about the pathophysiology of ITP, that future studies should look at the effects of injecting anti-muskin antibodies on immune responses when TIM-3 expression is limited to specific cell types, and other methods such as flow cytometry are needed to evaluate the role of T Cell Immunoglobulin Mucin Domain-3 in newly diagnosed children with ITP and its possible relation to disease outcome.

## Conclusions

This study investigated TIM-3 role in newly diagnosed children with ITP and its potential correlation with disease outcomes. The findings suggested that TIM-3 may play a critical role in ITP pathogenesis, potentially influencing the immune response and platelet destruction in these patients. Elevated TIM-3 expression may represent a therapeutic approach against ITP in children. This suggested that TIM-3 might be a valuable marker for predicting the severity of the disease. Additionally, improving or restoring TIM-3 function could have offered a new approach to treating ITP in children.

The positive correlation between TIM-3 levels and platelet counts at diagnosis and at remission suggests that TIM-3 may play a role in regulating platelets in ITP patients. Higher TIM-3 levels could have alleviated autoimmune diseases. Additionally, a negative correlation between TIM-3 levels and the bleeding score suggested that higher TIM-3 levels might have helped reduce bleeding severity in these patients. Overall, the study revealed the potential of TIM-3 as a marker for disease monitoring and offered a deeper understanding of the immune processes affecting ITP.

## Data Availability

Data are available upon reasonable request from the corresponding author.

## Abbreviations

CBC: Complete Blood Count
CD8+: Cytotoxic
ELISA: Enzyme-Linked Immunosorbent Assay
ESR: Erythrocyte Sedimentation Rate
IgG: Immunoglobulin G
ITP: Immune Thrombocytopenia
IVIG: Intravenous Immunoglobulin
TIM-3: T Cell Immunoglobulin Mucin Domain-3
TPO: Two Thrombopoietin
WBC: White Blood Cell
